# Nosocomial outbreak caused by disinfectant-resistant *Serratia marcescens* in an adult intensive care unit, Hungary, February to March 2022

**DOI:** 10.2807/1560-7917.ES.2024.29.26.2300492

**Published:** 2024-06-27

**Authors:** Adrienn Hanczvikkel, Ákos Tóth, Irén Anna Kopcsóné Németh, Orsolya Bazsó, Lőrinc Závorszky, Lilla Buzgó, Virág Lesinszki, Dániel Göbhardter, Erika Ungvári, Ivelina Damjanova, Attila Erőss, Ágnes Hajdu

**Affiliations:** 1 National Center for Public Health and Pharmacy (NNGYK), Budapest, Hungary; 2 ECDC Fellowship Programme, Public Health Microbiology path (EUPHEM), European Centre for Disease Prevention and Control (ECDC), Stockholm, Sweden; 3 North-Pest Central Hospital – Military Hospital (ÉPC-HK), Budapest, Hungary

**Keywords:** *Serratia marcescens*, matched case-control studies, disinfectant resistance, bloodstream infection, nosocomial outbreak, intensive care unit, infection control measures, whole genome sequencing, environmental sampling

## Abstract

In 2022, an outbreak with severe bloodstream infections caused by *Serratia marcescens* occurred in an adult intensive care unit (ICU) in Hungary. Eight cases, five of whom died, were detected. Initial control measures could not stop the outbreak. We conducted a matched case–control study. In univariable analysis, the cases were more likely to be located around one sink in the ICU and had more medical procedures and medications than the controls, however, the multivariable analysis was not conclusive. Isolates from blood cultures of the cases and the ICU environment were closely related by whole genome sequencing and resistant or tolerant against the quaternary ammonium compound surface disinfectant used in the ICU. Thus, *S. marcescens* was able to survive in the environment despite regular cleaning and disinfection. The hospital replaced the disinfectant with another one, tightened the cleaning protocol and strengthened hand hygiene compliance among the healthcare workers. Together, these control measures have proved effective to prevent new cases. Our results highlight the importance of multidisciplinary outbreak investigations, including environmental sampling, molecular typing and testing for disinfectant resistance.

**Key public health message**

**What did you want to address in this study?**

*Serratia marcescens* is a bacterium that can survive for long periods on objects and surfaces in hospitals and, if not controlled by stringent cleaning and disinfection and good hand hygiene, can cause serious outbreaks. We investigated an outbreak of bloodstream infections caused by *S. marcescens* in an adult intensive care unit (ICU) to understand how this bacterium spread among the patients and what can be done to prevent similar outbreaks.

**What have we learnt from this study?**

*Serratia marcescens* could not be eliminated by the surface disinfectant originally used. The bacterium could have survived in or around a sink in the main ICU room, and staff could have moved it into the patient zones via cleaning wipes. Occasional breaches of hand hygiene rules by healthcare workers and a more frequent care needed by the cases probably contributed to the spread of the bacterium from the environment to the patients.

**What are the implications of your findings for public health?**

Multiple approaches that include analyses of patient data and various microbiological methods, are the best way to identify factors leading to an outbreak. In case of *S. marcescens*, it is important to consider that it may be resistant to surface disinfectants. Replacing the ineffective disinfectant, reviewing environmental cleaning and disinfection protocols and strengthening compliance to hand hygiene contributed to the containment of this outbreak.

## Background

*Serratia marcescens*, a Gram-negative facultative anaerobic opportunistic pathogen, is the fifth most commonly reported causative agent of nosocomial outbreaks worldwide [1] and the ninth most frequent microorganism in bloodstream infections (BSI) associated with intensive care unit (ICU) in the European Union/European Economic Area (EU/EEA) countries [2]. In Hungary, outbreak reporting is mandatory by legislation [3], therefore healthcare providers shall immediately report clusters or outbreaks of any healthcare-associated infections (HAIs) to the public health authorities (PHA). Six outbreaks of healthcare-associated BSI caused by *S. marcescens* were reported in Hungary between 2016 and 2021, five of them in ICUs [4]. The most serious outbreak occurred in a COVID-19 ICU in 2020, with 30 symptomatic BSI cases, 26 of them fatal. In the other five outbreaks, altogether 20 symptomatic BSI cases and three deaths were reported (2016: 4 cases, 2017: 3 cases, 2018: 5 cases and 1 death, 2019: 2 cases, 2021: 6 cases and 2 deaths).

Healthcare-associated infections caused by *S. marcescens* are often linked to contaminated surfaces with the bacterium persisting in the hospital environment [5-10]. *Serratia marcescens* has a wide temperature (5–40°C) and pH (pH 5–9) range for growth [11]. Although the bacterium prefers moist environments (e.g. washbasins, taps, drains, liquid soap dispensers, air-conditioning systems or nebulizers), it can survive up to 2 months on dry surfaces [5,12,13], up to 20 days in anaerobic conditions or in conditions with low oxygen levels [14] and contaminate surface disinfectants, especially in case of inappropriate use (e.g. lower concentration or contact time than required) [15]. The ability of *S. marcescens* to adapt to disinfectants containing quaternary ammonium compound (QAC) has been shown in several studies, but there are fewer studies on QAC resistance from real-world settings [15]. Hands of healthcare workers (HCWs), with or without gloves, are a common vehicle of *S. marcescens* transmission, but a wide range of contaminated medical devices (e.g. bronchoscopes, laryngoscopes, ventilation equipment), medications (e.g. injectable solutions in multi-dose vials, prefilled syringes) and disinfectants have also been implicated [11]. Thus, detection of the reservoirs and possible routes of transmission is essential in containing outbreaks.

## Outbreak detection

In early February 2022, *S. marcescens* was detected in a patient with BSI in a multidisciplinary section of an adult ICU (ICU-1) of a tertiary teaching hospital in Hungary. When another case was detected 4 days later, the infection control nurse responsible for HAI surveillance in ICU-1 alerted the head of the hospital Department of Hospital Hygiene (DHH). The hospital DHH implemented infection control measures. On 1 March, the outbreak with seven cases by then, was reported to the PHA.

A detailed retrospective outbreak investigation was initiated by the hospital and the National Center for Public Health and Pharmacy (NCPHP) on 31 March 2022. In this paper, we aimed to describe the full investigation of the outbreak and interventions to contain it.

## Methods

### Setting

**Figure 1:**
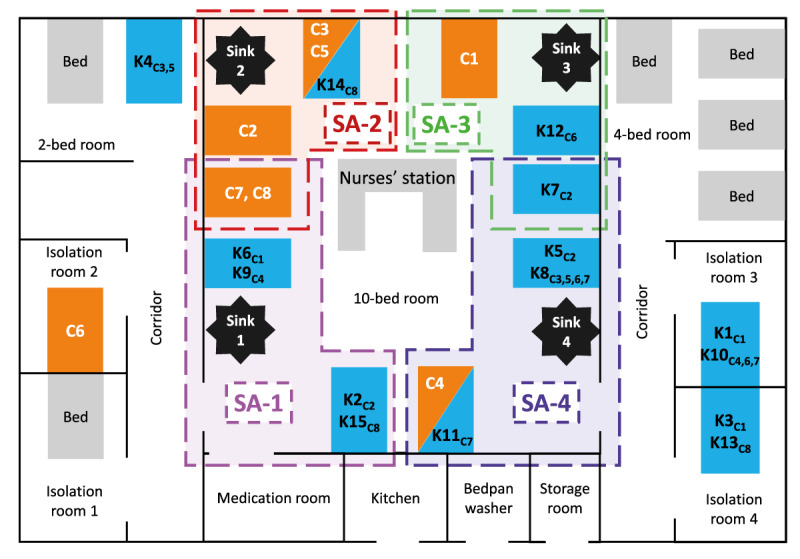
Illustration of hospital rooms and bed locations in an outbreak of *Serratia marcescens* in an intensive care unit, Hungary, February–March 2022

The ICU-1 had 20 beds in seven rooms (Figure 1). In the main 10-bed room of ICU-1, four taps and sinks were used for handwashing and occasionally, during renal replacement therapy, as a water source and for discarding the effluent fluid. In the outbreak period, the cardiac section with 10 beds was not operational, so patients undergoing cardiac surgery received post-operative care in the multidisciplinary section. The median daily bed occupancy rate of ICU-1 was 75% (range: 40‒95%). The daily number of nurses per patient varied between one and three. Nineteen of the 43 nurses worked overtime. Typically, two and occasionally three physicians were assigned to a patient. Physicians of ICU-1 also served as anaesthesiologists in the operating theatres. The relevant medical and cleaning procedures and processes used in ICU-1 are detailed in Supplementary material 1.

C1–C8: beds of confirmed cases highlighted in orange; K1–15: beds of matched controls highlighted in blue, the subscripts show the cases they were matched to; SA-1‒4: sink areas, including the three beds closest to a given sink, highlighted with dashed lines.

### Epidemiological investigation

We collected basic demographic, clinical and administrative data and information on bed location from all patients treated in ICU-1 between 9 February and 21 March 2022. A detailed description of the unit and the work schedule of nurses and doctors were obtained.

We conducted a matched case–control study to identify the source or vehicle of the infection. We defined a confirmed case as a patient with *S. marcescens* detected from blood culture and treated in ICU-1 between 9 February and 21 March 2022. Controls were selected among *Serratia*-negative patients in ICU-1, and they were individually matched to cases on time in ICU-1 (admission to ICU-1 at least 24 h before the first positive blood culture was taken from the matched case, in consideration of the probable exposure period). All ICU-1 patients meeting the inclusion criteria for controls were enrolled in the case–control study. We collected the following exposure data covering 24 h before the first positive blood culture result of a given case and its matched controls: invasive devices (e.g. arterial, central venous and peripheral venous catheters, endotracheal and nasogastric tubes, ventricular, subdural and wound drains), medical and imaging procedures (e.g. blood transfusion, bronchoscopy, computed tomography, haemodialysis, surgery, X-ray), medications (e.g. antacids, antimicrobials, diuretics, electrolytes, heart and nervous system medications, non-steroidal anti-inflammatory drugs administered either intravenously or through a nasogastric tube) and bed location in ICU-1. To investigate the effect of bed location in relation to sinks in ICU-1, we created dichotomous variables named sink area 1, 2, 3 and 4, including the three beds closest to a given sink (Figure 1). To assess the effect of care intensity in the 24 h exposure period investigated, the total number of invasive devices in place, medical and imaging procedures and medication events were counted per patient and summed into a discrete variable frequency of care.

#### Data analysis

Dichotomous variables were compared using Fisher’s exact test. To compare the age distribution and the mean of frequency of care variable among cases and controls, Shapiro-Wilk test and two-sample t-test were used. Univariable conditional logistic regression was used to calculate matched odds radios (mOR) with 95% confidence intervals (CI) and with p values to estimate the association between potential exposures and *S. marcescens* BSI. For exposure variables where the number of discordant pairs of one type was zero, we applied the Woolf-Haldane-Anscombe correction [16]: two hypothetical matched case–control pairs were added to the data to increase the number of discordant case–control pairs of both types by one. Exposure variables with significant p values (p < 0.05) in the univariable analysis were included in the multivariable conditional logistic regression model. The statistical calculations were performed with R v4.1.3 [17], using packages of epiR, Epistats, reshape and survival.

### Microbiological analyses

During the outbreak period, enhanced microbiological screening was implemented among ICU-1 patients. Throat and groin swabs, scrapings of skin and, in case of ventilated patients, tracheal secretions were taken on admission and twice a week. Samples for blood culture were taken immediately when a patient showed signs or symptoms of a systemic infection. Other targeted clinical samples (bronchial wash, cannula or wound sample) were taken if the patient showed signs of infection. In early April, the hospital DHH took environmental samples in ICU-1 from sinks, surfaces of ventilators, ultrasound machines, perfusers and cleaning equipment (impregnated, reusable cleaning wipes, cleaning buckets, floor mops) using moistened sterile cotton swabs, by thoroughly wiping a 100 cm^2^ surface, if possible, in several directions. For sampling the drains, a sterile cotton swab was inserted to a depth of 5 cm in the drain and circular sweeps were performed. The samples were analysed at the clinical microbiology laboratory of the hospital. Samples were inoculated into cook meat broth (CMB), first incubated at 35°C for 24 h and thereafter at room temperature for further 24 h. No neutralising solution was used. After incubation, 50 µL of the CMB suspension was spread onto blood agar and MacConkey agar plates (bioMérieux Inc., Marcy-l'Étoile, France) and incubated at 35°C for 24 h. Isolates were identified with MALDI-TOF mass spectrometry (MALDI-TOF MS, Bruker Daltonics Inc., Billerica, the United States (US)). In early August 2022, microbiological sampling of the cleaning equipment used in ICU-1 was repeated.

Antimicrobial susceptibility testing of *S. marcescens* isolates was done against piperacillin-tazobactam, ceftazidime, ceftriaxone, cefepime, ertapenem, imipenem, meropenem, ciprofloxacin, amikacin, gentamicin, tobramycin and trimethoprim-sulfamethoxazole using a disc diffusion method according to the European Committee on Antimicrobial Susceptibility Testing (EUCAST) guidelines [18,19] at the accredited Healthcare-associated Infections and Antimicrobial Resistance National Reference Laboratory (HAI-AMR-NRL) of the NCPHP.

The surface disinfectant used in ICU-1 contained two quaternary ammonium compounds (alkyl dimethylbenzyl ammonium chloride 9.7%, CAS: 68424–85–1 [20] and poly-1-hexamethylene biguanide hydrochloride 0.92%, CAS: 27083–27–8, 32289–58–0 [21]), hereinafter referred to as QAC-SfD. Using a broth microdilution method [19], we made a twofold dilution series of the concentrated QAC-SfD (stock solution) and determined the minimum concentration of the disinfectant that inhibited the growth of the *S. marcescens* isolates. This minimum inhibitory concentration (MIC) of an isolate was compared to the concentration of the in-use, working solution of QAC-SfD, and was expressed as its percentage, hereinafter referred to as QAC-SfD IC%. The formula for the calculation is:


$$QAC-SfD\;IC\%=\frac{minimum\;inhibitory\;concentration\;of\;QAC-SfD\;against\;a\;given\;isolate\;}{concentration\;of\;the\;in-use,\;working\;solution\;of\;QAC-SfD}\times 100$$

Unlike antimicrobials, there are no susceptibility breakpoints available for disinfectants. Based on the review of John M Boyce [15], we defined a QAC-SfD resistant isolate as one not inactivated by the in-use, working concentration of the QAC-SfD (i.e. QAC-SfD IC% > 100%). The term QAC-SfD tolerant was used when the QAC-SfD IC% of an isolate was < 100% but higher than the value of non-outbreak *S. marcescens* isolates from other patients in ICU-1. We used American Type Culture Collection (ATCC https://www.atcc.org/) 25922 *Escherichia coli* isolate as a quality control isolate.

We conducted pulsed-field gel electrophoresis (PFGE) on all *S. marcescens* isolates according to the quality control manual for the standardised PFGE technique issued by the Centers for Disease Control and Prevention (CDC, Atlanta, US) [22]. Digital images of gels were analysed with Fingerprinting II Informatix Software (Bio-Rad Laboratories, Hercules, US). Levels of similarity were calculated with the Dice coefficient. The unweighted pair group method with arithmetic averages (UPGMA) was used for cluster analysis of the PFGE band patterns. Isolates with less than 85% similarity were classified into different PFGE types and named as per the Hungarian national PFGE database.

For whole genome sequencing (WGS), libraries were prepared with the Nextera DNA Flex library preparation kit and sequenced with Illumina Miseq 2 × 150 bp pair-end kit (Illumina, San Diego, US). Sequencing results were accepted if the following quality indicators were fulfilled: N50 > 100 kb and average depth of sequencing coverage at least 50-fold. Using SeqSphere+ 8.5 software (Ridom GmbH, Münster, Germany), we conducted genomic data analysis and de novo assembly by Velvet v1.1.04 in Pipeline Mode with default settings. Clonal relationships were investigated by core genome multilocus sequence typing (cgMLST) stable scheme of SeqSphere+ 8.5 (2692 target genes, cluster distance threshold of 12 allele differences). For comparative genomic analysis, we used the Rapid Annotation Subsystem Technology (RAST) v2.0 server [23], ResFinder v4.1 and MyDbFinder v2.0 online tools of the Center Genomic Epidemiology (CGE), Technical University of Denmark (DTU) (http://genomicepidemiology.org/services/) by default settings.

## Results

### Epidemiological investigations

**Figure 2:**
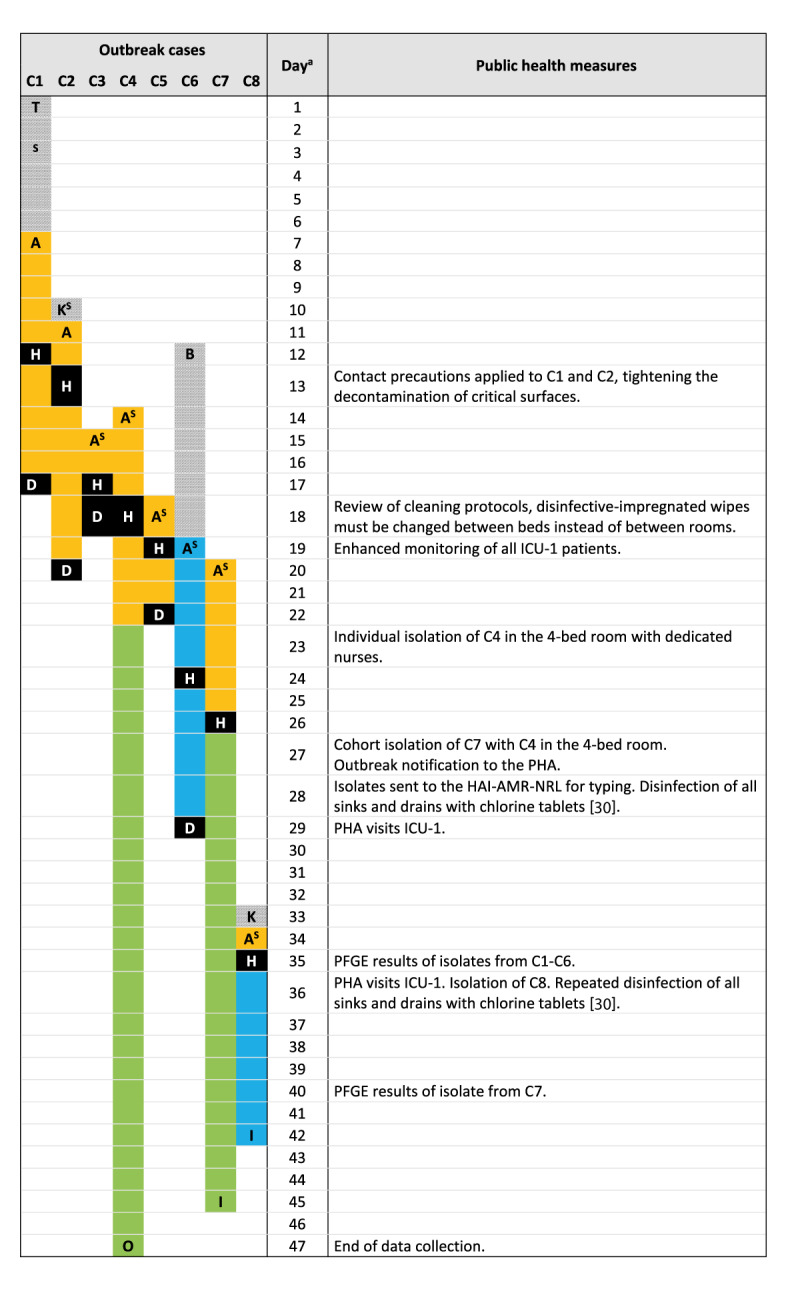
Timeline of an outbreak of *Serratia marcescens* in an intensive care unit, Hungary, February–March 2022

We detected eight cases (4.9%) among the 162 patients treated in ICU-1 during the outbreak period. The timeline of the outbreak and control measures are presented in Figure 2.

A: admission to ICU-1; B: admission to Department B; D: death; HAI-AMR-NRL: Healthcare-associated Infections and Antimicrobial Resistance National Reference Laboratory; H: sampling of the first *Serratia marcescens*-positive blood culture of the patient; I: intrahospital transfer; ICU-1: intensive care unit 1; K: admission to Department K; NA: not applicable; O: ongoing treatment; PHA: public health authority; PFGE: pulsed-field gel electrophoresis; T: admission to Department T; ^S^: surgery since hospital admission.

^a^ Day 1 is the day of admission of Case 1 to the hospital.

The orange colour corresponds to main 10-bed room in ICU-1, green to 4-bed room in ICU-1, blue to single-bed isolation room in ICU-1 and grey to department of the hospital other then ICU-1.

All cases were male, ranging in age from their mid-30s to mid-70s and underwent surgical interventions in seven different operating theatres by different surgical and anaesthesia teams at the hospital prior to admission to ICU-1. The cases had an illness of the central nervous system, an acute or chronic cardiovascular condition or severe burns. Four cases were transferred to ICU-1 from other departments of the hospital. No cases were detected in other units of the hospital.

The median time between admission to ICU-1 and detection of *S. marcescens* in a blood culture was 3 days (range: 1–6 days). Significantly more cases (5/8) died than other ICU-1 patients (22/154) (p < 0.01). Six cases had symptom onset on a weekend or 1 day after a weekend. The number of HCWs on duty did not differ between weekends and weekdays and no procedure was performed more commonly on weekends. No doctor or nurse was assigned to more than three cases.

Fifteen patients met the criterion of a matched control. Five cases were matched with three controls and three cases with two controls. The location of beds of cases and matched controls is shown in Figure 1. Case 6 was the only case staying in a single-bed isolation room at symptom onset. No significant association was found between being a case and age, sex or being exposed to any invasive devices, medical and imaging procedures, antacids, antimicrobials, diuretics, electrolytes, heart medications, nervous system medications, non-steroidal anti-inflammatory drugs or other medications and substances, as presented in Table 1 and in Supplementary material 3 (full analysis). The mean frequency of care for cases was 36 (standard deviation (SD): 3; range: 31–41), significantly higher (p < 0.01) than for controls (28; SD: 7; range: 17–46). In the univariable analysis, cases were significantly more likely to be in sink area 2 than controls and for increase of one unit in frequency of care, the odds of being a case significantly increased by a factor of 1.35, but in the multivariable analysis, none of the exposure variables was significant (Table 1).

**Table 1:** Number of exposed cases and controls, crude and adjusted matched odds ratios of statistically significant exposures in a univariable model and results of multivariable analysis in an investigation of an outbreak of *Serratia marcescens* in an intensive care unit, Hungary, February–March 2022^a^

Exposure	Exposed cases (n = 8)	Exposed controls (n = 21)	Univariable analysis			Multivariable analysis		
			Crude mOR	95% CI	p value	Adjusted mOR	95% CI	p value
Sink area 2	5	1	10.82	1.3–92	0.03	4.88	0.49–48	0.18
Frequency of care score	8	21	1.35^b^	1.03–1.80	0.03	1.30^b^	0.98–1.70	0.07

CI: confidence interval; mOR: matched odds ratio.

^a^ p values < 0.05 were considered statistically significant.

^b^ For increase of one unit in frequency of care.

### Microbiological and environmental findings

In total, 789 samples were taken from 162 patients in ICU-1 during the outbreak period. *Serratia marcescens* was detected from 18 patient samples: from all eight cases and from two other patients (Patient 1 and 2) (Table 2). From these two other patients, *S. marcescens* was not isolated from blood. From four cases (C1, C4, C6 and C7), *S. marcescens* was detected from more than one sample type. Seventeen patient isolates were submitted to the HAI-AMR-NRL for confirmation and further testing. From Case 1, *S. marcescens* was first detected from a tracheal secretion sample 5 days before detection from blood culture, but the tracheal isolate was not sent to the HAI-AMR-NRL. A total of 34 environmental samples were taken, and four *S. marcescens* isolates were detected from three of them, as presented in Supplementary material 2.

**Table 2:** Characterisation of *Serratia marcescens* isolates from cases and hospital environment in an outbreak investigation in an intensive care unit, Hungary, February–August 2022 (n = 22)

Sample origin	Sample ID	Sample type	Day of sampling^a^	Acquired antimicrobial resistance	PFGE	QAC-SfD IC%	CT
Case 1	TRA1	Tracheal secretion	7	NA	NA	NA	NA
Case 1	HC1	Blood	12	TOB	A	156	809
Case 2	HC2	Blood	13	TOB	A	156	809
Case 2	HC2b	Blood	17	TOB	A	NT	NT
Case 3	HC3	Blood	17	TOB	A	156	809
Case 4	HC4	Blood	18	TOB	A	156	809
Case 4	TRA4	Tracheal secretion	27	ND	B	5	813
Case 4	S4	Skin scrapings	27	ND	B	NT	NT
Case 4	HC4b	Blood	28	TOB	A	NT	NT
Case 4	B4	Bronchial washing	21	ND	B	NT	NT
Case 5	HC5	Blood	19	TOB	A	78	809
Case 6	HC6	Blood	24	TOB	A	156	809
Case 6	W6	Swab from wound	26	TOB	A	NT	NT
Case 7	TRA7	Tracheal secretion	24	TOB	A	NT	NT
Case 7	HC7	Blood	27	TOB	A	78	809
Case 8	HC8	Blood	35	TOB	A	156	809
Patient 1^b^	CAN-P1	Cannula	25	TOB	C	20	812
Patient 2^b^	TRA-P2	Tracheal secretion	31	CIP, CN, TOB	C	NT	NT
Blue-coloured cleaning wipe 1	BW1	Environmental swab	63	ND	D	78	810
Blue-coloured cleaning wipe 2	BW2	Environmental swab	63	CIP, TOB	E	312	811
Red-coloured cleaning bucket	RB	Environmental swab	188	ND	D	78	810
Green-coloured cleaning bucket	GB	Environmental swab	188	CIP, TOB	E	312	811

CIP: ciprofloxacin; CN: gentamicin; CT: complex type; ICU: intensive care unit; ID: identification code; NA: isolate not available; ND: not detected; NT: not tested; PFGE: pulsed-field gel electrophoresis profile; QAC-SfD: surface disinfectant used in ICU-1; QAC-SfD IC%: minimum inhibitory concentration of isolates against the surface disinfectant used in ICU-1 compared to the concentration of the in-use, working solution (100%); TOB: tobramycin.

^a^ Day of sampling, where Day 1 is the day of admission of Case 1 to the hospital.

^b^ Patient in ICU-1 with *Serratia marcescens* detected from another sample type than blood.

None of the *S. marcescens* isolates were multidrug-resistant, based on a previous definition of multidrug resistance [24,25]. Case isolates from blood cultures (HC1-HC8) were indistinguishable by PFGE (type A) and tolerant or resistant against QAC-SfD (QAC-SfD IC%: 78–156%). The four environmental isolates belonged to two different PFGE types (BW1 and RB to type D and BW2 and GB to type E), but all were tolerant or resistant against QAC-SfD (QAC-SfD IC%: 78–312%) (Table 2). Isolates from Patient 1 (CAN-P1) and Patient 2 (TRA-P2) were different from the isolates from blood cultures by PFGE (type C) and were considered unrelated to the outbreak. Isolates TRA4 (PFGE type B) and CAN-P1 (PFGE type C) were QAC-SfD sensitive (QAC-SfD IC%: 5–20%).

**Figure 3:**
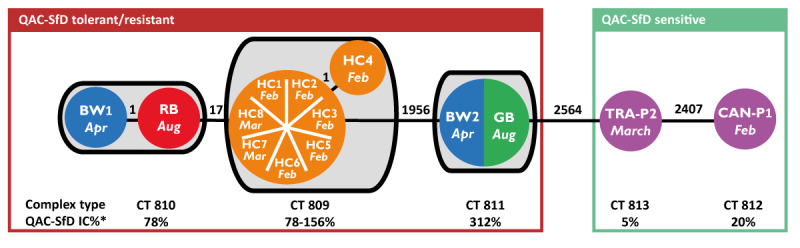
Minimum spanning tree of *Serratia marcescens* isolates from an outbreak investigation in an intensive care unit, Hungary, February–August 2022 (n = 14)

Based on the results of the antimicrobial susceptibility testing and PFGE, 14 isolates were whole genome sequenced (Figure 3). Isolates from blood cultures belonged to complex type (CT) 809 and clustered (0–1 allele differences). Two isolates from the cleaning equipment (BW1 and RB) were of CT 810 and closely related to the outbreak strain with 17–19 allele differences. All other isolates (BW2, GB, TRA4 and CAN-P1) were distinct (≥ 1956 allele differences).

BW1: isolate from an environmental swab; BW2: isolate from an environmental swab; CAN-P1: isolate from cannula of patient 1; cgMLST: core genome multilocus sequence typing; CT: complex type; GB: isolate from an environmental swab; HC1-HC8: isolates from blood cultures of cases 1–8; ICU-1: intensive care unit 1; QAC-SfD: surface disinfectant used in ICU-1; QAC-SfD IC%: minimal inhibitory concentration of isolates against the surface disinfectant used in ICU-1 compared with the manufacturer’s recommendation (100%); RB: isolate from an environmental swab; TRA-P2: isolate from a tracheal secretion sample of patient 2.

The circles represent *Serratia marcescens* isolates. Numbers on the lines show the allele differences between the isolates based on the stable cgMLST (2692 genes) scheme of Ridom SeqSphere+ (cluster distance threshold 12). The dates show the months of detection of *Serratia marcescens*.

Using the RAST bioinformatics tool, we identified additional genes in the HC1-HC8 isolates and in the closely related ones from cleaning equipment (BW1 and RB) compared with two non-related isolates (TRA4 and CAN-P1). These genes are responsible for membrane transport (e.g. efflux systems [26]), biofilm production (e.g. *pgaABCD* operon [27]), degradation of aromatic compounds (e.g. hydroxyaromatic non-oxidative decarboxylase operon), stress response (detoxification, oxidative and osmotic stress response genes (*bet* operon)), formaldehyde (*formA* [28]) and heavy metal resistance (CHASRI) [29]. With one exception, none of these genes was found in the genome of the isolates recovered from screening samples (TRA4, CAN-P1). Genes responsible for resistance against formaldehyde, silver/copper and for the degradation of aromatic compounds were not present in the genome of the unrelated isolates from cleaning equipment (BW2, GB) either. Detailed results of the comparative genomic analysis are presented in Supplementary material 4.

## Outbreak control measures

The hospital DHH took several control measures to contain the outbreak (Figure 2). Generic control measures, such as (i) placing cases under contact precautions, (ii) isolation of cases, (iii) enhanced monitoring (on-site visits in ICU-1, review of the medical and nursing documentation as well as follow-up of the clinical course of all ICU-1 patients by an infection control nurse on a daily basis) and microbiological screening of ICU-1 patients, (iv) reviewing and modifying the environmental cleaning protocol (disinfection-impregnated wipes had to be used per bed instead of per room), (v) disinfection of sinks and drains with chlorine tablets [30] and (vi) reinforcing awareness on the importance of hand hygiene.

The local PHA visited ICU-1 on two occasions in March (Figure 2). During the first visit, the local PHA identified deficiencies mainly relating to labelling of solutions and how solutions were accessed. These deficiencies were corrected by the second visit. As no new cases were detected, the outbreak was closed at the end of March 2022 by the local PHA.

After the detection of QAC-SfD resistant *S. marcescens* from the environment in August 2022, the hospital DHH replaced the QAC-SfD with another disinfectant containing two active agents: C12-C18 alkyl benzyl dimethyl ammonium chloride, CAS: 68391–01–5 [31] and N-(3-aminopropyl)-N-dodecylpropane-1,3-diamine, CAS: 2372–82–9 [32]. This surface disinfectant requires a shorter contact time (5 min) than the previous one (30 min). The cleaning equipment was also replaced in August, the after-use disinfection protocol of the cleaning equipment was tightened, and regular microbiological sampling of the cleaning equipment was introduced.

While the HCW were not formally interviewed about their adherence to rules of hand hygiene and of sink use, members of the outbreak investigation team from ICU-1 noted that rules of sink use were largely respected, however, they saw the need to reinforce hand hygiene compliance from time to time. The importance of hand hygiene compliance in the prevention of HAIs was emphasised to the staff of ICU-1 by the hospital DHH.

## Discussion

In this outbreak, five of the eight detected cases died, which is unusual, but not unprecedented. Case fatalities of *S. marcescens* BSI vary between 14 and 85% [33,34]. We did not identify any genes in the outbreak strain suggesting an increased pathogenicity. Considering the mode of transmission, our hypothesis is that the QAC-SfD-resistant *S. marcescens* was transferred from sink area 2 into the patient zones by the cleaning equipment during the routine disinfection process and from these contaminated surfaces to the patients and possibly from infected patients to other patients by the hand of HCWs.

For *S. marcescens*, opposed to several other Enterobacterales, colonisation is typically transient, and it is the environmental exposure that poses a risk of bacteraemia [35]. Several studies showed that *S. marcescens* can persist lengthily on surfaces and in water sources, including taps or tap water [6,36,37]. Transmission of pathogenic bacteria from drains to the area around tap, onto the devices stored there or to the hands of healthcare professionals has been seen in several studies [13,38-40]. If an outbreak strain is detected from environmental samples and accurate sampling times are known, the Modified Causal Analysis/Diagnosis Decision Information System (CADDIS) tool can be used to evaluate the likelihood of a causal relationship between sinks and patients [41]. In this outbreak in ICU-1, repeated cleaning and disinfection as part of the initial infection prevention and control measures may have limited the spread and hampered the detection of the pathogen from the environment. However, other studies demonstrated that even after disinfection of drains and sinks, Gram-negative bacteria could still be detected [8]. Complete elimination of QAC-resistant bacteria from the environment may be further complicated by possible co-resistance and cross-resistance to antimicrobials [42].

Practical issues around the daily use of QAC-SfD in ICU-1 could have contributed to the development of QAC-SfD tolerance or resistance in *S. marcescens*. According to the manufacturer's instructions, the required contact time of QAC-SfD was 30 min. Surfaces to be disinfected should have remained wet for this time, which was challenging e.g. on vertical surfaces. Inappropriate contact time or concentration may contribute to triggering stress responses in bacteria that induce changes in their cell envelope or efflux pump activity, resulting in higher environmental tolerance [43]. Mechanisms of QAC tolerance in *Serratia* species include innate bacterial cell wall structure, changes in cell membrane structure and function, efflux pumps, biofilm formation and QAC degradation [15,44]. Results of our RAST analysis showed that the outbreak strain and the closely related environmental isolates (BW1, RB) had genes encoding the functions to survive in the hospital environment and to adapt to QAC disinfectants [15]. Due to the presence of CHASRI in their genome, these isolates had the potential to become resistant against copper or silver-based disinfectants as well by a single-point mutation either in their *cus* and/or *sil* genes [29].

Our WGS analysis confirmed that isolates from the cases’ blood cultures clustered. Case isolates and some isolates from the cleaning equipment (BW1, RB) were closely related, despite the 6 months between the isolation dates. Two other, genetically distinct *S. marcescens* isolates (BW2, GB) were QAC-SfD tolerant/resistant, which further highlights the importance of stringent cleaning and disinfection routines with effective agents as a key control measure.

The possibility of *S. marcescens* transmission before ICU-1 admission (e.g. in operating theatres) was unlikely because no cases were detected elsewhere in the hospital. Frequency of care or frequent manipulation, as a proxy for increased exposure, has been documented as a risk factor in nosocomial outbreaks [45]. In ICU-1, hand hygiene breaches could have concerned several HCWs and vascular catheter care, leading to catheter-associated BSI, one of the most common healthcare-associated infections in ICUs [46-49]. Of note, the nurse-to-patient ratio in ICU-1 did not always reach 1:1 as recommended for ventilated patients [50], indicating a suboptimal workload of nurses. Increased workload has a negative impact on hand hygiene compliance, even in ICUs [51,52]. Immediate transfer of surviving cases to isolation rooms with dedicated nursing staff was hampered by the prior (unrelated) transient closure of the cardiac section of ICU-1, affecting the functioning of the multidisciplinary section.

Our findings confirmed the usefulness of environmental monitoring. The relevant legal regulation [53] and recommendations from the NCPHP [54] include environmental monitoring as a task for hospitals and the PHA, however, implementation appears to be heterogeneous due to financial and access constraints. This highlights the need for a systematic evaluation of environmental sampling practices in HAI outbreaks in Hungary.

Despite the prompt identification of the outbreak and the implementation of control measures by the hospital DHH, the NCPHP recommended reviewing notification routines and improving the timeliness of notification. To strengthen compliance with local protocols to prevent, among others, catheter-associated BSI, the hospital DHH and Department of Internal Quality Control have set up a system of monthly clinical audits during which they select five patients per unit and systematically review documentations and procedures on site.

Considering the limitations of our investigation, we chose to focus on potential exposures within 24 h of symptom onset based on the general concept of device-associated HAIs (i.e. an HAI in a patient with a relevant device that was used within 48 h before onset of infection) and descriptive data of the cases. Nonetheless, the interval between the insertion or use of invasive devices and the onset of bacteraemia can be longer than 24 h, even several days [55,56], therefore we may have missed some important exposures that occurred earlier.

Due to limited resources, no environmental samples were taken during the active phase of the outbreak and the number of environmental samples taken later was suboptimal. No particular attention was paid to sink area 2 and the surrounding surfaces and objects stored next to the sink. In August, only the sampling of the cleaning equipment was repeated. Samples of soap, alcohol-based hand rubs, dispensers and patients’ skin disinfectants used before surgery and invasive procedures were not taken. Sampling of hands of HCWs during the active phase of the outbreak to collect more evidence about possible transmission routes could have been informative, yet it was not implemented at the time.

Finally, we could not investigate the expression or functionality of the various virulence or resistance genes identified in the genome of *S. marcescens* isolates. Thus, we could not conclude if the overexpression of the efflux pump genes found in all isolates contributed to the elevated QAC-SfD IC% values [26,57,58]. However, our conclusions are consistent with the results of our phenotypic investigations and with data available in the literature.

## Conclusion

To summarise, this outbreak emphasises (i) the high-level adaptability of *S. marcescens* to the hospital environment, (ii) the need for the prudent selection and use of disinfectants with a periodic review of environmental cleaning and disinfection protocols, (iii) the necessity of environmental screening and, if justified, testing for disinfectant-resistance tailored to the given context, (iv) the importance of compliance with infection prevention and control precautions during patient care, including hand hygiene, and (v) the value of multidisciplinary approaches in nosocomial outbreak investigations.

## Supplementary Data

901000 Supplementary Material
