# Supplementary material for: Nosocomial outbreak caused by disinfectant-resistant Serratia marcescens in an adult intensive care unit, Hungary, February to March 2022
Source: Euro Surveill. 2024 Jun 27;29(26):2300492. doi: 10.2807/1560-7917.ES.2024.29.26.2300492 (PMC11212457; doi:10.2807/1560-7917.ES.2024.29.26.2300492)
Supplement: Supplementary Material [file 23-00492_HANCZVIKKEL_Supplement.pdf]

This supplementary material is hosted by Eurosurveillance as supporting information alongside the article [Nosocomial outbreak caused by disinfectant-resistant *Serratia marcescens* in an adult intensive care unit, Hungary, February to March 2022], on behalf of the authors, who remain responsible for the accuracy and appropriateness of the content. The same standards for ethics, copyright, attributions and permissions as for the article apply. Supplements are not edited by Eurosurveillance and the journal is not responsible for the maintenance of any links or email addresses provided therein.

Supplementary material 1

Relevant medical and cleaning procedures and processes in the ICU-1

The hospital had a protocol for insertion and maintenance of central (CVC) and peripheral vascular catheters (PVC) based on the latest national recommendations issued by the chief medical officer [1]. The hospital's CVC insertion bundle components included hand hygiene before CVC insertion, maximal sterile barriers and aseptic technique, skin antisepsis with nationally authorised, min. 70% alcohol-based skin disinfectant (preferably containing chlorhexidine), optimal catheter site selection and appropriate dressing. The hospital's CVC maintenance bundle components included daily review of CVC necessity, procedures for catheter site dressing monitoring/changes, hand hygiene before any catheter manipulation, catheter site care at dressing changes with nationally authorised, min. 70% alcohol-based skin disinfectant (preferably containing chlorhexidine), and disinfection of catheter injection ports before each manipulation. Similar insertion and maintenance bundles were in place for PVC and pulmonary artery catheters. Protocols were also available for the insertion and maintenance of peripheral artery catheters and of ventricular pressure monitoring devices.

Sterillium (BODE Chemie, Hamburg, Germany) was used as alcohol-based hand rub and individual dispensers were available at each bed in ICU-1. Skinman Scrub (Ecolab Inc, Saint Paul, the United States (US)) was used as antimicrobial soap, available at each sink. Hygienic hand washing (as opposed to hand rubbing with alcohol-based handrub) was done according to indications defined by the World Health Organization (WHO) [2]. The hospital had a protocol for the cleaning and disinfection of dispensers that included weekly surface disinfection with wipes, and manual or mechanical cleaning and disinfecting of the dosing pumps whenever a disposable container was replaced.

Environmental cleaning and disinfection were regulated in a targeted protocol for ICUs in the hospital. The cleaning personnel of ICU-1 was permanent staff. Fluorescent markers were used on a quarterly basis to assess cleanliness. In ICU-1, QAC-SfD was used for the disinfection of surfaces and sinks. The cleaning staff routinely diluted the original concentrate to the manufacturer's recommended concentration using a sensor-based forced-dilution device. The cleaning staff used impregnated, reusable cleaning wipes to clean furniture, equipment and devices. The wipes were impregnated with the QAC-SfD diluted as described above, and taken to the place of cleaning in sealed boxes. After use, the wipes were laundered and then re-impregnated. Until the outbreak, the impregnated wipes had been changed between rooms. Due to the outbreak, the hospital modified the cleaning protocol and requested to change wipes between beds.

References:

1. National Public Health Centre (NNK). Az országos tisztifőorvos módszertani levele az érkatéterrel összefüggő véráramfertőzések megelőzésére. [Methodological letter from the national medical officer on vascular catheter-related bloodstream infections prevention.]: Budapest: NNK; 2019.

Available in Hungarian from: https://www.antsz.hu/data/cms89792/Modszertani_level_az_erkateterrel_osszefuggo_veraramfertozesek_megelozesere.pdf.

2. WHO Guidelines Approved by the Guidelines Review Committee. WHO Guidelines on Hand Hygiene in Health Care: First Global Patient Safety Challenge Clean Care Is Safer Care. Geneva: World Health Organization Copyright © 2009, World Health Organization.; 2009. Available from: https://iris.who.int/bitstream/handle/10665/44102/9789241597906_eng.pdf?sequence=1.

This supplementary material is hosted by Eurosurveillance as supporting information alongside the article [Nosocomial outbreak caused by disinfectant-resistant *Serratia marcescens* in an adult intensive care unit, Hungary, February to March 2022], on behalf of the authors, who remain responsible for the accuracy and appropriateness of the content. The same standards for ethics, copyright, attributions and permissions as for the article apply. Supplements are not edited by Eurosurveillance and the journal is not responsible for the maintenance of any links or email addresses provided therein.

Supplementary material 2

Results of positive environmental samples from the intensive care unit 1, associated to the bloodstream-infection outbreak caused by disinfectant-resistant *Serratia marcescens* in Hungary, February-March 2022.

Sampling month	Sample	Microbiology results
April	blue-coloured, impregnated cleaning wipe after use in the 10-bed room, used for the cleaning of furniture, equipment and devices	***Serratia marcescens_1*** (Sample ID: BW1)<br>***Serratia marcescens_2*** (Sample ID: BW2)
April	mop_1	*Staphylococcus epidermidis*<br>*Staphylococcus oralis*
April	mop_2	*Bacillus cereus*
April	sink tap 1 (10-bed room)	*Staphylococcus epidermidis*<br>*Staphylococcus hominis*<br>*Staphylococcus pasteuri*
April	sink drain (Isolation room 2)	*Pseudomonas oleovorans*<br>*Stenotrophomonas maltophilia*
April	sink drain 1 (10-bed room)	*Achromobacter xylosoxidans*<br>*Citrobacter freundii*<br>*Staphylococcus epidermidis*<br>*Pseudomonas monteilii*
April	sink drain (Medication room)	*Staphylococcus epidermidis*
April	sink tap (Isolation room 2)	*Chryseobacterium sp.*<br>*Pseudomonas oleovorans*
April	ultrasound device head (10-bed room)	*Staphylococcus epidermidis*<br>*Staphylococcus capitis*<br>*Staphylococcus warneri*
April	ventilator (Isolation room 2)	*Staphylococcus epidermidis*
April	washbowl (Isolation room 2)	*Staphylococcus epidermidis*<br>*Staphylococcus hominis*<br>*Staphylococcus warneri*
August	blue-coloured cleaning bucket (ICU-1)	*Achromobacter sp.*<br>*Staphylococcus hominis*
August	blue-coloured bucket_2 (Storage room)	*Trichoderma sp.*
August	blue-coloured impregnating bucket (Storage room)	*Paracoccus yeei*
August	blue-coloured bucket_1 (Storage room)	*Bacillus licheniformis*

August	green-coloured bucket (Storage room)	*Bacillus thuringiensis*<br>*Moraxella osloensis*<br>*Paenibacillus alvei*<br>*Staphylococcus hominis*<br>*Staphylococcus petrasii*
August	green-coloured cleaning bucket (ICU-1), used for the cleaning of the kitchen furniture	*Achromonas sp.*<br>***Serratia marcescens*** (Sample ID: GB)
August	red-coloured cleaning bucket (ICU-1), used for the cleaning of toilets, urinals and adult nappy bins	*Burkholderia cepacia*<br>***Serratia marcescens:*** (Sample ID: RB)
August	red-coloured bucket (Storage room)	*Bacillus sp.*<br>*Micrococcus luteus*
August	yellow-coloured bucket_1 (Storage room)	*Aerococcus viridans*<br>*Staphylococcus haemolyticus*<br>*Staphylococcus hominis*
August	yellow-coloured bucket_2 (Storage room)	*Achromobacter sp.*<br>*Bacillus licheniformis*<br>*Bacillus mycoides*<br>*Brevundimonas diminuta*<br>*Cellulomonas sp.*<br>*Pseudomonas aeruginosa*<br>*Pseudomonas putida*<br>*Pseudomonas stutzeri*<br>*Sphingobacterium thalphophylum*<br>*Stenotrophomonas maltophilia*

This supplementary material is hosted by Eurosurveillance as supporting information alongside the article [Nosocomial outbreak caused by disinfectant-resistant *Serratia marcescens* in an adult intensive care unit, Hungary, February to March 2022], on behalf of the authors, who remain responsible for the accuracy and appropriateness of the content. The same standards for ethics, copyright, attributions and permissions as for the article apply. Supplements are not edited by Eurosurveillance and the journal is not responsible for the maintenance of any links or email addresses provided therein.

Supplementary material 3

Number of exposed cases and controls, crude and adjusted matched odds ratios in univariable and multivariable models in an investigation of an outbreak of *Serratia marcescens* in an intensive care unit, Hungary, February–March 2022 [a, b]

Exposure	Exposed cases (total n = 8)	Exposed controls (total n = 21)	Univariable analysis			Multivariable analysis		
			Crude mOR	95% CI	p value	Adjusted mOR	95% CI	p value
Bed location								
Sink area 1	2	4	1.46	0.23–9.1	0.69	Not applicable		
**Sink area 2**	**5**	**1**	**10.82**	**1.3–92**	**0.03**	4.88	0.49-48	0.18
Sink area 3	1	2	1.50	0.14–16	0.74	Not applicable		
Sink area 4	1	7	0.32	0.04–2.8	0.31			
**Frequency of care**	**8**	**21**	**1.35**	**1.03–1.8**	**0.03**	1.30	0.98-1.7	0.07
Invasive devices								
Arterial catheter – brachial	1	10	0.22	0.03–1.8	0.16	Not applicable		
Arterial catheter – radial	6	9	5.27	0.60–46	0.13			
Arterial catheter – total number	8	19	1.59	0.10–24	0.74			
Central venous catheter – jugular	3	9	0.88	0.16–4.9	0.89			
Central venous catheter – subclavian	6	12	1.62	0.29–9.0	0.58			
Central venous catheter – total number	8	21	3.00	0.42–21	0.27			
Peripheral venous catheter	2	3	1.56	0.26–9.4	0.63			
PiCCO device	1	2	1.73	0.10–30	0.71			
Endotracheal tube	7	15	2.41	0.26–23	0.44			
Nasogastric tube	8	19	1.87	0.18–19	0.60			
Ventricular or subdural drain	2	2	2.45	0.34–18	0.73			
Wound drain – total number	4	12	0.58	0.21–1.6	0.29			
Medical and imaging procedures								
Blood transfusion	4	7	2.70	0.71–10	0.15	Not applicable		
Bronchoscopy	1	1	2.45	0.15–40	0.53			
Haemodialysis	1	1	3.00	0.19–48	0.44			
Surgery	1	1	2.45	0.15–40	0.53			
Wound dressing	2	0	1.00	0.14–7.1	1.00			
Electrocardiogram (ECG)	0	4	0.36	0.04–3.2	0.36			
Computed tomography (CT)	3	3	2.20	0.59–8.2	0.24			
Ultrasonography	4	4	6.00	0.65–57	0.11			
X-ray	5	8	3.30	0.75–15	0.11			
Antacids								
Pantoprazole brand A	4	7	0.91	0.28–3.0	0.88	Not applicable		

Pantoprazole brand B	2	8	0.71	0.22–2.3	0.56	
Famotidine	4	6	1.20	0.56–2.6	0.64	
Antimicrobials						
Amoxicillin-clavulanic acid	1	0	2.56	0.23–29	0.45	Not applicable
Cefazolin	3	2	1.57	0.66–3.7	0.31	
Ciprofloxacin	1	1	2.72	0.41–18	0.30	
Erythromycin	1	0	2.35	0.46–12	0.30	
Gentamicin	2	0	4.96	0.49–50	0.18	
Meropenem	1	1	1.13	0.45–2.9	0.80	
Piperacillin-tazobactam	4	4	1.70	0.76–3.8	0.20	
Vancomycin	1	1	2.45	0.15–40	0.53	
Fluconazole	1	1	1.73	0.34–8.8	0.51	
Diuretics						
Furosemide	2	10	0.61	0.27–1.4	0.24	Not applicable
Spironolactone brand C	1	2	0.84	0.18–3.9	0.82	
Spironolactone brand D	1	1	1.27	0.20–8.1	0.80	
Electrolytes						
Calcium	2	2	2.71	0.38–19	0.32	Not applicable
Potassium chloride	2	3	1.29	0.36–4.6	0.70	
Multi-electrolyte solution	8	14	4.92	0.53–45	0.16	
Heart medications						
Norepinephrine	3	2	1.86	0.36–9.5	0.45	Not applicable
Metopropol	1	1	3.00	0.42–21	0.27	
Bisoprolol	1	0	3.00	0.47–19	0.24	
Amiodarone	1	1	1.00	0.24–4.2	1.00	
Digoxin	1	1	1.27	0.20–8.1	0.80	
Urapidil	2	1	2.32	0.60–9.0	0.23	
Magnesium and potassium aspartate	2	0	3.43	0.66–18	0.14	
Propranolol	1	0	3.00	0.47–19	0.24	
Nervous system medications						
Propofol	4	11	0.91	0.20–4.1	0.90	Not applicable
Levetiracetam	2	3	1.39	0.55–3.5	0.49	
Quetiapine	2	6	0.88	0.45–1.7	0.71	
Alprazolam	3	1	4.10	0.54–31	0.17	
Zopiclone	1	0	3.00	0.25–36	0.39	
Midazolam	1	0	2.56	0.23–29	0.45	
Sufentanil	2	2	2.18	0.22–22	0.51	
Tramadol	2	2	2.52	0.62–10	0.20	
Rocuronium bromide	1	0	2.56	0.23–29	0.45	
Clonazepam	1	3	0.78	0.30–2.0	0.61	
Non-steroidal anti-inflammatory drugs (NSAID)						
Metamizole sodium	3	8	0.96	0.52–1.8	0.88	Not applicable
Acetylsalicylic acid	2	3	2.30	0.31–17	0.42	
Ibuprofen	4	7	1.34	0.81–2.2	0.26	
Paracetamol	8	14	2.45	0.91–6.6	0.08	
Diclofenac	1	0	3.00	0.47–19	0.24	

Other medications and substances						
Enoxaparin sodium (anticoagulant)	6	16	0.87	0.25–3.1	0.83	Not applicable
Insulin (antidiabetic)	3	5	1.77	0.33–9.5	0.51	
Metoclopramide (antiemetic)	4	6	1.25	0.72–2.2	0.44	
Rosuvastatin (antilipemic)	1	0	3.00	0.25–36	0.39	
Lactulose (laxative)	2	2	3.00	0.52–18	0.22	
Acetylcysteine (mucolytic)	1	1	1.26	0.57–2.8	0.58	
Mannitol (osmotic agent)	3	0	4.54	0.53–39	0.17	
CT contrast dye	1	1	2.45	0.15–40	0.53	
Hydrocortisone (steroid hormone)	3	0	3.50	0.77–16	0.10	
Mixture of B vitamins	1	3	0.95	0.31–2.9	0.93	

CI: confidence interval; mOR: matched odds ratio; PiCCO: Pulse index Continuous Cardiac Output device.

[a] p values < 0.05 were considered statistically significant.

[b] Medications to which only controls were exposed are not presented in the table, but the frequency of care variable included these as well. Urinary catheter was not included in the univariable analysis as an exposure variable, as all cases and controls had one, but was included in the frequency of care variable.

This supplementary material is hosted by Eurosurveillance as supporting information alongside the article [Nosocomial outbreak caused by disinfectant-resistant *Serratia marcescens* in an adult intensive care unit, Hungary, February to March 2022], on behalf of the authors, who remain responsible for the accuracy and appropriateness of the content. The same standards for ethics, copyright, attributions and permissions as for the article apply. Supplements are not edited by Eurosurveillance and the journal is not responsible for the maintenance of any links or email addresses provided therein.

Supplementary material 4

Results of the comparative genomic analysis of *S. marcescens* strains isolates from cases and hospital environment in an outbreak investigation in an intensive care unit, Hungary, February–August 2022

Strains were isolated from: HC1-HC8 –blood culture samples of the cases, TRA4 – tracheal tube secretion of case 4 with a *S. marcescens* different from the outbreak strain, CAN-P1 – cannula of a patient from ICU-1 with a *S. marcescens* different from the outbreak strain, BW1 and BW2 – blue-coloured, impregnated, reusable cleaning wipe, RB – a red cleaning bucket, GB – a green cleaning bucket.

**Comparative results from Rapid Annotation Subsystem Technology (RAST) v2.0 server [1].**				**Sample ID of *S. marcescens* strains**				
**category**	**subcategory**	**subsystem**	**role**	**HC1 - HC8**	**CAN-P1**	**TRA4**	**BW2 GB**	**BW1 RB**
Regulation and Cell signaling	Quorum sensing and biofilm formation	Biofilm Adhesin Biosynthesis	Biofilm PGA outer membrane secretin *PgaA*	Y	N	N	Y	Y
Regulation and Cell signaling	Quorum sensing and biofilm formation	Biofilm Adhesin Biosynthesis	Biofilm PGA synthesis N-glycosyltransferase *PgaC* (EC 2.4.-.-)	Y	N	N	Y	Y
Regulation and Cell signaling	Quorum sensing and biofilm formation	Biofilm Adhesin Biosynthesis	Biofilm PGA synthesis auxiliary protein *PgaD*	Y	N	N	Y	Y
Regulation and Cell signaling	Quorum sensing and biofilm formation	Biofilm Adhesin Biosynthesis	Biofilm PGA synthesis deacetylase *PgaB* (EC 3.-)	Y	N	N	Y	Y
Metabolism of Aromatic Compounds	Anaerobic degradation of aromatic compounds	Hydroxyaromatic decarboxylase family	Hydroxyaromatic non-oxidative decarboxylase protein B, *bsdB* (EC 4.1.1.-)	Y	N	N	N	Y
Metabolism of Aromatic Compounds	Anaerobic degradation of aromatic compounds	Hydroxyaromatic decarboxylase family	Hydroxyaromatic non-oxidative decarboxylase protein C, *bsdC* (EC 4.1.1.-)	Y	N	N	N	Y

Metabolism of Aromatic Compounds	Anaerobic degradation of aromatic compounds	Hydroxyaromatic decarboxylase family	Hydroxyaromatic non-oxidative decarboxylase protein D, *bsdD* (EC 4.1.1.-)	Y	N	N	N	Y
Metabolism of Aromatic Compounds	Metabolism of central aromatic intermediates	Salicylate and gentisate catabolism	Maleate cis-trans isomerase (EC 5.2.1.1)	Y	N	Y	Y	Y
Metabolism of Aromatic Compounds	Metabolism of central aromatic intermediates	Salicylate and gentisate catabolism	Salicylate hydroxylase (EC 1.14.13.1)	Y	N	N	N	Y
Stress Response	Detoxification	Tellurite resistance: Chromosomal determinants	Tellurite resistance protein *TehA*	Y	N	N	Y	Y
Stress Response	Osmotic stress	Synthesis of osmoregulated periplasmic glucans	*OpgC* protein	Y	N	N	Y	Y
Stress Response	Oxidative stress	Glutaredoxins	Cell wall endopeptidase, family M23/M37	Y	N	N	Y	Y
Stress Response	Oxidative stress	Oxidative stress	Redox-sensitive transcriptional activator *SoxR*	Y	N	N	Y	Y
**Membrane Transport**	Protein and nucleoprotein secretion system, Type IV	Conjugative transfer	Conjugative transfer protein *TrbB*	Y	N	N	Y	Y
**Membrane Transport**	Protein and nucleoprotein secretion system, Type IV	Conjugative transfer	Conjugative transfer protein *TrbC*	Y	N	N	Y	Y
**Membrane Transport**	Protein and nucleoprotein secretion system, Type IV	Conjugative transfer	Conjugative transfer protein *TrbD*	Y	N	N	Y	Y
**Membrane Transport**	Protein and nucleoprotein secretion system, Type IV	Conjugative transfer	Conjugative transfer protein *TrbE*	Y	N	N	Y	Y
**Membrane Transport**	Protein and nucleoprotein secretion system, Type IV	Conjugative transfer	Conjugative transfer protein *TrbF*	Y	N	N	Y	Y

**Membrane Transport**	Protein and nucleoprotein secretion system, Type IV	Conjugative transfer	Conjugative transfer protein *TrbG*	Y	N	N	Y	Y
**Membrane Transport**	Protein and nucleoprotein secretion system, Type IV	Conjugative transfer	Conjugative transfer protein *TrbI*	Y	N	N	Y	Y
**Membrane Transport**	Protein and nucleoprotein secretion system, Type IV	Conjugative transfer	Conjugative transfer protein *TrbJ*	Y	N	N	Y	Y
**Membrane Transport**	Protein and nucleoprotein secretion system, Type IV	Conjugative transfer	Conjugative transfer protein *TrbL*	Y	N	N	Y	Y
**Membrane Transport**	TRAP transporters	TRAP Transporter unknown substrate 9	TRAP transporter solute receptor, TAXI family precursor	Y	N	N	Y	Y
**Membrane Transport**	TRAP transporters	TRAP Transporter unknown substrate 9	TRAP-type uncharacterized transport system, fused permease component	Y	N	N	Y	Y
Virulence, Disease and Defense	Resistance to antibiotics and toxic compounds	Cobalt-zinc-cadmium resistance	Copper sensory histidine kinase *CusS*	Y	N	N	N	Y
Virulence, Disease and Defense	Resistance to antibiotics and toxic compounds	Cobalt-zinc-cadmium resistance	Copper-sensing two-component system response regulator *CusR*	Y	N	N	N	Y
Virulence, Disease and Defense	Resistance to antibiotics and toxic compounds	Cobalt-zinc-cadmium resistance	Probable Co/Zn/Cd efflux system membrane fusion protein	Y	N	N	Y	Y
Virulence, Disease and Defense	Resistance to antibiotics and toxic compounds	Copper homeostasis	*CopG* protein	Y	N	N	N	Y
Virulence, Disease and Defense	Resistance to antibiotics and toxic compounds	Copper homeostasis	Copper resistance protein B	Y	N	N	N	Y
Virulence, Disease and Defense	Resistance to antibiotics and toxic compounds	Copper homeostasis	Multicopper oxidase	Y	N	N	N	Y

Comparative results from Resfinder v4.1 and MyDBFinder v2.0 analysis, Center for Genomic Epidemiology (CGE), Technical University of Denmark (DTU) http://genomicepidemiology.org/services/				HC1 - HC8	CAN-P1	TRA4	BW2 GB	BW1 RB
Virulence, Disease and Defense	Resistance to antibiotics and toxic compounds	Formaldehyde resistance	formaldehyde dehydrogenase, *formA* [2]	Y	N	N	N	Y
Virulence, Disease and Defense	Resistance to antibiotics and toxic compounds	Copper Homeostasis and Silver Resistance Island	*sil* operon *pco* operon [3]	Y	N	N	N	Y

References:

1. Aziz RK, Bartels D, Best AA, DeJongh M, Disz T, Edwards RA, et al. The RAST Server: Rapid Annotations using Subsystems Technology. BMC Genomics. 2008;9(1):75.
2. Kümmerle N, Feucht HH, Kaulfers PM. Plasmid-mediated formaldehyde resistance in Escherichia coli: characterization of resistance gene. Antimicrobial Agents and Chemotherapy. 1996;40(10):2276-9.
3. Hanczvikkel A, Füzi M, Ungvári E, Tóth Á. Transmissible silver resistance readily evolves in high-risk clone isolates of Klebsiella pneumoniae. Acta Microbiologica et Immunologica Hungarica. 2018;65(3):387-403.
